# Macrophages in Chronic Rejection: The Shapeshifters Behind Transplant Survival

**DOI:** 10.3390/biology15020162

**Published:** 2026-01-16

**Authors:** Ahmed Uosef, Jacek Z. Kubiak, Rafik M. Ghobrial

**Affiliations:** 1 Transplant Immunology, The Houston Methodist Research Institute, Houston, TX 77030, USA; 2 Department of Surgery, The Houston Methodist Hospital, Houston, TX 77030, USA; 3 Dynamics and Mechanics of Epithelia Group, Faculty of Medicine, Institute of Genetics and Development of Rennes, University of Rennes, CNRS, UMR 6290, 35000 Rennes, France; 4 Laboratory of Molecular Oncology and Innovative Therapies, Department of Oncology, Military Institute of Medicine, 04-141 Warsaw, Poland

**Keywords:** macrophages, chronic rejection, macrophage polarization (M1/M2), NLRP3 inflammasome, NF-κB signaling, tissue remodeling

## Abstract

**Background:** Organ transplant offers patients a second chance at life, yet chronic rejection remains a formidable barrier to long-term success. Unlike the instantaneous storm of acute rejection, chronic rejection is a slow, unremitting process that silently remodels vessels, scars tissues, and diminishes graft function. At the center of this process are macrophages, immune “shapeshifters” that can heal or harm depending on their cues. **Methods:** This manuscript systematically reviews and synthesizes the current evidence from experimental studies and clinical observations, as well as molecular insights, to unravel how macrophages orchestrate chronic rejection. It travels over macrophage origins alongside their dynamic polarization into pro-inflammatory (M1) or pro-repair yet fibrotic (M2) states. The discussion integrates mechanisms of recruitment, antigen presentation, vascular injury, and fibrosis, while highlighting the molecular pathways (NF-κB, inflammasomes, STAT signaling, metabolic rewiring) that shape macrophage fate. **Results:** Macrophages play a central role in chronic rejection. Resident macrophages, once tissue peacekeepers, amplify inflammation, while recruited monocyte-derived macrophages fuel acute injury or dysfunctional repair. Together, they initiate transplant vasculopathy through cytokines, growth factors, and matrix metalloproteinases, slowly narrowing vessels and starving grafts. Donor-derived macrophages, often overlooked, act as early sentinels and long-term architects of fibrosis, blurring the line between donor and host immunity. At the molecular level, macrophages lock into destructive programs, perpetuating a cycle of inflammation, vascular remodeling, and scarring. **Conclusions:** Macrophages are not passive bystanders but pivotal decision makers in chronic rejection. Their plasticity, while a source of pathology, also opens therapeutic opportunities. Emerging strategies like macrophage-targeted drugs, immune tolerance approaches, gene and exosome therapies currently offer ways to reprogram these cells and preserve graft function. By shifting the macrophage narrative from saboteurs to guardians, transplantation medicine may transform chronic rejection from an inevitability into a preventable complication, extending graft survival from fleeting years into enduring decades.

## Simple Summary

Long-term survival of transplanted organs is limited by chronic rejection, a slow and progressive process that damages blood vessels and leads to loss of organ function. This review explains how macrophages, a type of immune cell with highly adaptable functions, play a central role in driving chronic rejection. Macrophages can originate from both the donor organ and the transplant recipient and can switch between inflammatory and tissue-repair programs in response to local signals. In chronic rejection, these cells often promote ongoing inflammation, vascular remodeling, and fibrosis. Understanding how macrophages contribute to chronic rejection may help guide the development of new therapies aimed at preserving transplanted organs and improving long-term transplant outcomes.

## 1. Introduction to Chronic Rejection—The Long Game of the Immune System

When a person receives a new organ, the moment feels like a victory, a second chance at life. Nevertheless, in the background, a quiet, relentless battle often begins. Unlike the dramatic flare of acute rejection, which can strike within days or weeks, chronic rejection is a slow burn. It unfolds over months or even years, steadily wearing down the transplanted organ.

Chronic rejection remains a major cause of late graft failure across solid organ transplantation, with incidence and clinical manifestations varying by organ type. In kidney transplantation, chronic antibody-mediated rejection accounts for a substantial proportion of graft losses beyond the first post-transplant year, while in heart and lung transplantation, transplant vasculopathy and chronic lung allograft dysfunction are the principal determinants of long-term survival. Liver transplantation shows comparatively lower rates of classical chronic rejection, reflecting a more tolerogenic immune environment [1,2]. Importantly, chronic rejection in solid organ transplantation is distinct from immune complications following hematopoietic stem cell (bone marrow) transplantation, where graft-versus-host disease represents a systemic donor immune response against recipient tissues rather than a localized host-versus-graft process.

Doctors can see its fingerprints in the body’s plumbing: blood vessels in the graft thicken from the inside out, a process called transplant vasculopathy. This narrowing chokes the organ’s blood supply, much like corrosion inside a pipe. Over time, scar tissue builds up, healthy cells falter, and the organ’s function fades [3,4].

At the center of this slow-motion drama are the macrophages, the immune system’s shapeshifters. In a healthy body, they are caretakers, clearing debris and helping tissues heal. However, in chronic rejection, these same cells can turn into relentless agitators, bridging the body’s fast-acting innate defenses with the precise, long-memory attacks of adaptive immunity [5].

When provoked, macrophages release inflammatory molecules such as TNF-α and IL-1β, which act like alarm bells summoning more immune cells to the scene. They release growth factors such as PDGF and TGF-β that coax smooth muscle cells to proliferate within the vessel wall [6]. They even deploy molecular scissors, enzymes called matrix metalloproteinases, to cut through the tissue’s scaffolding, reshaping blood vessels in ways that often make things worse [7].

The result is a tug-of-war between repair and destruction. Despite decades of medical advances, chronic rejection remains one of the top reasons transplanted organs fail. For kidney recipients, it can mean returning to dialysis; for heart and lung recipients, it can mean the difference between years of life gained and a premature end. Understanding how macrophages drive this process is not just an academic puzzle; it is the key to keeping life-saving organs working for the long haul.

Chronic rejection is not a single pathological phenomenon but rather encompasses distinct immunological responses with unique clinical and histological signatures. According to the Banff classification, chronic rejection is broadly divided into **chronic** antibody-mediated rejection (cABMR) and chronic T cell–mediated rejection (cTCMR) [8]. In kidney transplantation, chronic antibody-mediated rejection (cABMR) is characterized by transplant glomerulopathy, multilayering of peritubular capillary basement membranes, persistent microvascular inflammation, and evidence of circulating donor-specific antibodies, often accompanied by C4d deposition or endothelial injury–associated molecular signatures [8,9]. In contrast, chronic T cell–mediated rejection (cTCMR) in the kidney is defined by sustained interstitial inflammation, tubulitis, and progressive interstitial fibrosis and tubular atrophy, reflecting chronic cellular immune activation rather than antibody-driven vascular injury.

Importantly, growing evidence indicates that macrophages contribute differently to these two forms of chronic kidney allograft rejection. In renal cABMR, macrophages are preferentially enriched within glomeruli and peritubular capillaries, where Fcγ receptor–mediated interactions with donor-specific antibodies promote endothelial injury, complement-independent inflammation, and chronic microvascular remodeling. These macrophage-driven processes are increasingly recognized as key contributors to progressive transplant vasculopathy and graft dysfunction in kidney allografts [10].

By contrast, in cTCMR, macrophages interact more closely with T cells and parenchymal cells, functioning as antigen-presenting cells and sources of pro-inflammatory and pro-fibrotic mediators that perpetuate cellular rejection and progressive tissue scarring [11]. Recognizing these macrophage-driven distinctions between humoral and cellular chronic rejection provides an essential framework for understanding the diverse mechanisms by which macrophages shape long-term graft injury and failure.

## 2. Macrophage Origins and Plasticity—Two Roads to the Same Cell

Not all macrophages in a transplanted organ originate from the same sources. Some have resided there since before the operation, quietly maintaining the tissue. Others are summoned in from the recipient’s bloodstream, like reinforcements answering a distress call. Together, they form a complex network of immune cells that can either protect the graft or quietly dismantle it over time.

Resident macrophages are the homebodies of the immune world. Born from embryonic precursors, they take up permanent residence in tissues and can live there for years [12]. In a healthy organ, these cells act as peacekeepers, clearing debris, dampening unnecessary immune responses, and facilitating repairs. However, transplantation changes their environment overnight. The shock of surgery, immune mismatches, and the stress of new blood flow can flip their switch. Instead of calming things down, they can start pumping out pro-inflammatory signals like TNF-α, IL-1β, and IL-6 [13]. These resident cells are also skilled at antigen presentation, meaning they can alert the adaptive immune system and escalate the conflict.

Monocyte-derived macrophages, in contrast, are the first responders. When a graft is injured, the tissue releases chemical “SOS” signals, including chemokines such as CCL2 (also known as MCP-1) and CXCL10. These act like homing beacons for circulating monocytes, which migrate into the graft and transform into macrophages [14]. Once inside, they can become either aggressive M1 macrophages or repair-oriented M2 macrophages, depending on the local signals they receive [14].

The two groups do not play identical roles. Resident macrophages are better at orchestrating long-term immune communication and can inadvertently promote fibrosis by overproducing TGF-β. Monocyte-derived macrophages, on the other hand, are the heavy hitters of acute inflammation, unleashing reactive oxygen species and inflammatory mediators that cause direct tissue injury.

**Figure 1:**
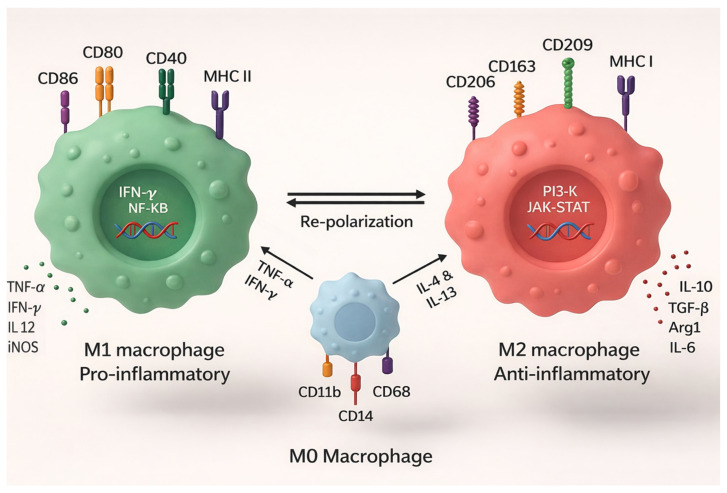
This illustration shows how naïve M0 macrophages, whether originating as long-lived resident cells or newly recruited monocytes, can polarize into either pro-inflammatory M1 or anti-inflammatory M2 phenotypes, reflecting the concepts described in Macrophage Origins and Plasticity. M1 macrophages, driven by signals such as IFN-γ and TNF-α, express markers including CD80, CD86, CD40, and MHC II and activate NF-κB–mediated pathways that promote inflammatory cytokine release. In contrast, M2 macrophages arise in response to IL-4 and IL-13, express markers such as CD206, CD163, and CD209, and rely on PI3K/JAK-STAT signaling to produce regulatory and pro-fibrotic mediators, such as IL-10 and. Clinically, high macrophage infiltration and expression of activation markers such as CD80/CD86 and MHC II are linked with increased inflammation, impaired graft function, and poor survival, whereas elevated expression of scavenger and repair-associated markers such as CD206 and CD163 often accompanies fibrotic remodeling and later graft deterioration. The bidirectional arrows highlight macrophage plasticity, emphasizing that both resident and monocyte-derived macrophages can shift between these states, an ability that profoundly influences graft inflammation, repair, and the progression of chronic rejection.

Macrophages also have a remarkable ability to reinvent themselves, a concept called polarization. In their M1 form, they act like soldiers in full combat mode, producing inflammatory cytokines that recruit more immune cells to attack the graft. In their M2 form, they produce growth factors such as TGF-β that promote healing, but can also lead to unwanted scarring [15]. The delicate balance between these states can determine whether a transplanted organ thrives or fails (Figure 1).

Although the M1/M2 paradigm provides a useful conceptual framework, macrophage activation in vivo spans a broad and continuous spectrum of functional states rather than discrete polarized phenotypes. In human pathology, particularly in transplanted organs, macrophages adopt context-dependent transcriptional programs shaped by tissue-specific cues, chronic immune stimulation, and metabolic stress. Single-cell RNA sequencing studies of human kidney allograft biopsies have revealed multiple macrophage subsets, including interferon-responsive inflammatory macrophages, fibrosis-associated remodeling macrophages, and the transitional populations exhibiting mixed inflammatory and reparative signatures, underscoring the limitations of the binary polarization model in chronic rejection [16,17].

The graft microenvironment further amplifies macrophage heterogeneity through hypoxia, altered nutrient availability, and sustained cellular stress. In murine transplant models, skewing macrophages toward pro-inflammatory phenotypes exacerbates vascular injury and accelerates chronic rejection, whereas promoting reparative or regulatory macrophage programs attenuates fibrosis and preserves graft function. Hypoxic conditions within transplanted organs promote metabolic reprogramming and stress-adaptive macrophage states that diverge from classical polarization patterns [18]. In parallel, single-cell analyses across human tissues identify conserved resident-like macrophage populations specialized in lipid handling, scavenging, and homeostatic maintenance, which can transition into pathogenic phenotypes under chronic inflammatory pressure [19]. These findings highlight macrophage plasticity as a dynamic, environmentally driven process and provide a more physiologically relevant framework for understanding macrophage-mediated injury in chronic rejection.

## 3. Recruitment and Activation—How Macrophages Get the Call

Once a transplanted organ begins showing signs of Stress, the immune system reacts almost instinctively. The blood stream macrophages are summoned to the graft and together with the resident macrophages are activated through a finely tuned network of chemical signals and danger cues.

The first invitations come from chemokines and cytokines such as CCL2 (MCP-1) and CXCL10. They create a chemical trail that monocytes can follow straight into the graft. Once they arrive, these cells transform into macrophages and choose their role in the battle.

**Figure 2:**
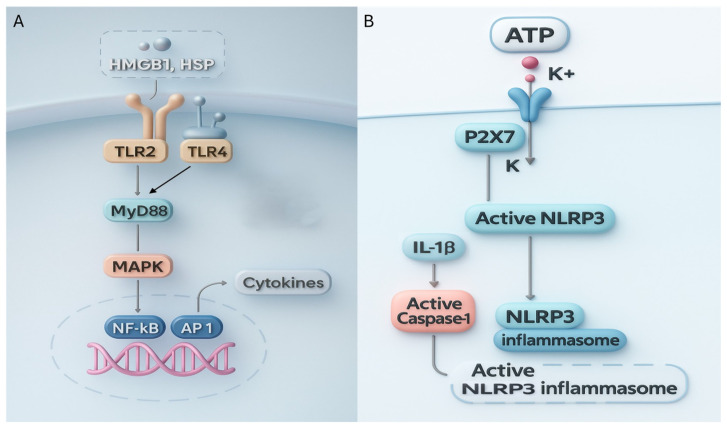
This schematic illustrates the molecular pathways by which macrophages detect and respond to danger signals in the transplanted organ, emphasizing how macrophages get the Call. (**A**) shows how DAMPs such as HMGB1 and heat shock proteins engage TLR2 and TLR4, triggering MyD88- and MAPK-dependent activation of NF-κB and AP-1, leading to pro-inflammatory cytokine production. (**B**) depicts ATP-mediated activation of the P2X7 receptor, promoting potassium efflux and initiating the NLRP3 inflammasome cascade, resulting in caspase-1 activation and IL-1β maturation. Together, these pathways highlight how macrophages rapidly sense tissue injury and amplify inflammatory signals within the graft.

Macrophages also carry pattern recognition receptors, tiny molecular “radar dishes” that pick up danger signals. When tissues are injured, they release damage-associated molecular patterns (DAMPs) such as HMGB1 and heat shock proteins. Toll-like receptors (such as TLR4) detect these signals and activate inflammatory programs through pathways such as NF-κB and MAPK [20]. Some sensors, such as NOD-like receptors, build entire molecular machines called inflammasomes. The NLRP3 inflammasome, for instance, activates caspase-1, which in turn produces IL-1β, a powerful spark for inflammation [21] (Figure 2).

Interestingly, NF-κB signaling, inflammasome activation, and metabolic reprogramming of macrophages operate as an interconnected regulatory network rather than independent pathways during chronic rejection. NF-κB provides the priming signal for inflammasome activation while simultaneously driving transcription of pro-inflammatory mediators, and glycolytic metabolic reprogramming sustains both processes by supporting energy demands and redox signaling. Conversely, mitochondrial dysfunction and reactive oxygen species further amplify inflammasome activity, reinforcing inflammatory loops within the chronically stressed graft. Persistent hypoxia and nutrient imbalance lock these pathways into self-sustaining circuits that favor ongoing inflammation, vascular remodeling, and fibrosis over resolution, positioning macrophages as integrative decision-makers in long-term graft injury [22,23,24].

Nevertheless, macrophages are not just independent operators; they work together with adaptive immunity. Through molecules like MHC-II and co-stimulatory partners CD80/CD86, macrophages can present antigens to T cells, effectively telling them, “Here is the intruder, attack here. They also secrete IL-12 and IL-23, which shape T-cell maturation and function. This keeps the immune response active long after the initial injury [25].

The graft environment adds another twist: hypoxia. A transplanted organ can experience oxygen shortages due to changes in blood flow or early damage. In low-oxygen conditions, macrophages stabilize HIF-1α, a protein that shifts their metabolism toward glycolysis and drives the production of factors such as VEGF, which, paradoxically can worsen tissue remodeling [26].

## 4. Macrophages in Transplant Vasculopathy—Remodeling Gone Wrong

One of the most destructive legacies of chronic rejection is transplant vasculopathy, a condition where the graft’s blood vessels are slowly reshaped in ways that impair function. If you think of the vasculature as the organ’s lifeline, vasculopathy is like a slow, unrelenting tightening of that lifeline.

Endothelial injury is often the first domino to fall. M1 macrophages, armed with reactive oxygen species and inflammatory cytokines, directly damage the endothelial cells that line the vessel walls [27]. Once those cells are weakened, the barrier becomes leaky, more immune cells flood in, and the cycle of injury accelerates.

Next, smooth muscle cell proliferation induced by growth factors such as PDGF and TGF-β, both produced by macrophages; smooth muscle cells migrate into the innermost layer of the vessel wall. This migration causes the intima to thicken, narrowing the blood vessel’s lumen. Blood flow slows, and the organ’s oxygen supply suffers.

**Figure 3:**
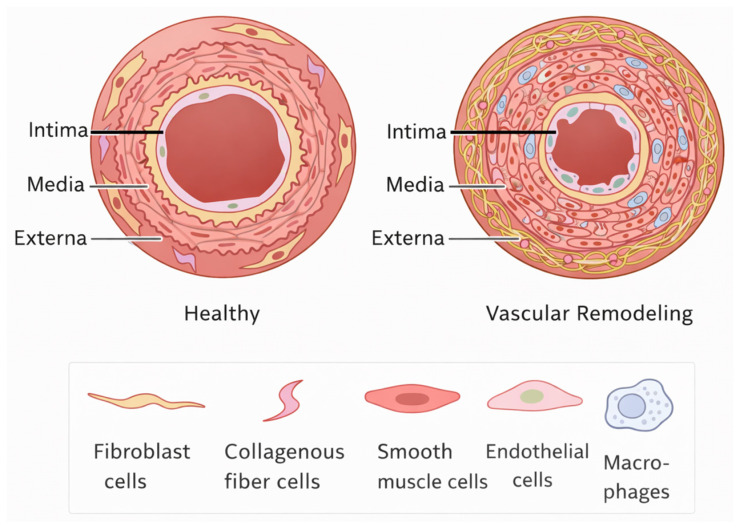
Schematic illustration of transplant vasculopathy in chronic rejection. The healthy vessel (**left**) shows intact endothelium, organized smooth muscle cells, and preserved vessel architecture. In contrast, vascular remodeling (**right**) is characterized by endothelial injury, macrophage infiltration, smooth muscle cell proliferation and migration into the intima, and dysregulated extracellular matrix deposition, resulting in intimal thickening and luminal narrowing. Worth mentioning that the intima consists of three distinct layers: Endothelium (the innermost layer), Basal lamina (supports the endothelium), and the subendothelial layer (collagen fibers mixed with SMCs and macrophages). Macrophage-derived inflammatory mediators, growth factors, and matrix-remodeling enzymes collectively drive progressive vascular fibrosis, impaired perfusion, and long-term graft dysfunction. The bottom panel summarizes the major cellular components involved in this process.

Finally, macrophages secrete matrix metalloproteinases (MMPs), which degrade the extracellular matrix [28]. Physiologically, matrix remodeling is part of regular repair. However, in chronic rejection, it becomes unbalanced, leading to the formation of disorganized scar tissue that affects the structural integrity of the vessels and the graft (Figure 3).

## 5. Donor Macrophages—The Silent Passengers That Shape Rejection

When an organ is transplanted, it brings along more than just tissue; it carries immune cells from the donor, including macrophages. These donor-derived macrophages are among the first to interact with the recipient’s immune system, making them early “passengers” that can either accelerate rejection or help the graft settle in.

### 5.1. Early Sentinels of Danger

Donor macrophages remain embedded in the transplanted organ at the time of surgery. As soon as blood flow is restored, these cells sense injury and stress signals, releasing cytokines such as TNF-α, IL-1β, and IL-6. This burst of inflammatory communication acts like a distress flare, alerting recipient immune cells and amplifying the danger signals that trigger acute rejection [29].

### 5.2. Antigen Presentation—Donor Cells as Teachers

Unlike passenger lymphocytes, donor macrophages are long-lived and present donor MHC molecules directly to recipient T cells. Through this direct antigen presentation, they serve as teachers of the recipient’s immune system, driving robust allogeneic T cell responses. In acute rejection, this pathway acts as a strong ignition source for the adaptive immune response [30].

### 5.3. Chronic Influence—Architects of Fibrosis

Even after the initial immune battle, donor macrophages can persist in the graft for weeks to months. These long-lived cells gradually switch roles, producing TGF-β and pro-fibrotic mediators that activate fibroblasts. Over time, this fosters scar tissue deposition and vascular remodeling, hallmarks of chronic rejection. Thus, donor macrophages may be temporary passengers, but their legacy can be long-lasting [31].

### 5.4. Cross-Talk with Recipient Cells

Donor macrophages do not act alone. They cross-communicate with recipient dendritic cells, endothelial cells, and infiltrating monocytes. This creates a complex immune network in which donor cells set the tone, and recipient cells amplify the response. Such cross-talk blurs the line between donor and host immunity, reinforcing rejection cascades.

### 5.5. Emerging Insights—Potential Targets

Recent studies suggest that selectively depleting donor macrophages before transplantation or modulating their activation state could reduce rejection severity. Therapies targeting CSF1R signaling (critical for macrophage survival) or promoting regulatory macrophage phenotypes are being explored as ways to disarm these hidden passengers before they ignite rejection.

The persistence and turnover of donor-derived macrophages vary substantially across organ types, influencing their long-term immunological impact. In organs such as the heart and lung, donor macrophages are progressively replaced by recipient-derived monocyte-derived macrophages over weeks to months, reflecting limited local self-renewal capacity and continuous immune cell recruitment [32]. In these settings, donor macrophages primarily shape early inflammatory signaling and antigen presentation, while recipient macrophages dominate chronic immune responses and tissue remodeling.

## 6. Fibrosis and Graft Dysfunction—When Healing Becomes Harm

Fibrosis is the immune system’s version of overzealous repair. It is like patching a hole in a wall with so much plaster that the wall becomes unusable. In chronic rejection, macrophages, especially those with an M2 phenotype, are major drivers of the process.

Through TGF-β signaling, M2 macrophages activate fibroblasts, the body’s collagen factories, and encourage them to churn out extracellular [33]. Collagen is not bad in itself; it gives tissues structure, but too many turns flexible tissues into rigid scars.

Macrophages also engage in cross-talk with fibroblasts via cytokines such as IL-13 and IL-4, pushing these cells into a constant state of activation. This interaction creates a feedback loop: fibroblasts make the tissue stiffer, stiffer tissue perpetuates immune stress, and immune stress keeps fibroblasts activated.

Sometimes, fibroblasts go one step further, transforming into myofibroblasts under the influence of macrophage-derived TGF-β. Myofibroblasts are scar tissue specialists, loaded with α-SMA and capable of producing vast amounts of extracellular matrix. Once they take over, the organ becomes progressively less functional [34].

## 7. Macrophage Molecular Pathways—The Signals Behind Their Shapeshifting

Macrophages are not just foot soldiers of the immune system; they are also bio-chemical command centers, constantly rewiring their internal pathways in response to signals from the transplanted organ. These molecular circuits determine whether a macrophage turns into an aggressor, fueling rejection, or a healer, trying to preserve tissue. In chronic rejection, unfortunately, many of these switches tilt toward destruction.

One of the most important switches is the NF-κB pathway. When macrophages detect damage signals, through the TLR4 receptor, NF-κB moves into the nucleus and activates a whole storm of inflammatory genes, TNF-α, IL-1β, and IL-6, that sustain chronic inflammation. This creates a feedback loop: the more the tissue suffers, the more danger signals it releases, and the harder NF-κB pushes the macrophage toward an M1 state [34].

Alongside NF-κB, the NLRP3 inflammasome acts like a molecular ignition system. When triggered by cellular stress, it activates caspase-1, which processes IL-1β and IL-18 into their active forms. These cytokines intensify the immune attack and contribute to vascular remodeling and fibrosis. In chronic rejection, overactive inflammasomes make macrophages potent amplifiers of injury.

Metabolism also guides macrophage fate. M1 macrophages rely on aerobic glycolysis—burning glucose quickly to sustain rapid-fire cytokine production. In contrast, M2 macrophages favor oxidative phosphorylation and fatty acid oxidation, fueling long-term repair programs. The balance between these metabolic states doesn’t just reflect macrophage identity; it helps lock it in. In chronic rejection, metabolic bias toward glycolysis keeps macrophages in a pro-inflammatory state [35].

Other signaling molecules fine-tune this polarization. STAT1 drives M1 behavior under the influence of IFN-γ, while STAT6 responds to IL-4 and IL-13 to reinforce M2 programming. MicroRNAs also play a part: miR-155 pushes macrophages toward inflammatory state, while miR-146b and miR-223 promote resolution and repair [36].

**Figure 4:**
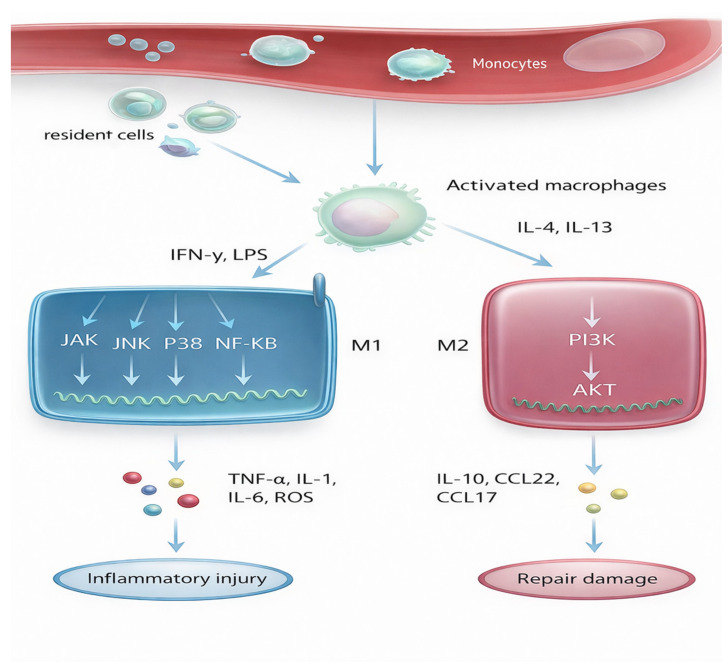
This figure illustrates the key molecular pathways that govern macrophage polarization Signals such as IFN-γ and LPS drive macrophages toward an M1 phenotype, activating pathways including JAK, JNK, p38, and NF-κB, which promote production of TNF-α, IL-1, IL-6, and ROS that contribute to inflammatory injury. In contrast, IL-4 and IL-13 stimulate PI3K–AKT signaling to induce M2 polarization, leading to secretion of IL-10 and chemokines such as CCL22 and CCL17 that support tissue repair. Together, the diagram highlights how distinct intracellular programs determine whether macrophages fuel graft damage or promote healing.

Finally, macrophages shape rejection via growth factor pathways. TGF-β secreted by M2 macrophages activates fibroblasts and promotes extracellular matrix deposition, leading to fibrosis. Meanwhile, PDGF signaling encourages smooth muscle cell proliferation in vessel walls, fueling transplant vasculopathy. Taken together, these pathways show macrophages as dynamic decision-makers. Macrophage signaling cascades—including NF-κB, inflammasomes, STAT pathways, metabolic programs, and microRNAs—form an integrated molecular script that can either defend or destroy the graft, reflecting a complex network of immune activation and regulation as highlighted in recent perspective analyses. In chronic rejection, the script is tragically tilted toward inflammation, scarring, and vascular injury. By targeting these molecular levers, future therapies may be able to rewrite macrophages’ role, turning them from saboteurs into guardians of long-term graft survival [37] (Figure 4).

## 8. Therapeutic Implications—Turning Knowledge into Action

If macrophages are central players in chronic rejection, then targeting them, or their signals, could shift the odds in a transplant’s favor. Macrophage-targeted therapies face important translational challenges in transplantation. Because macrophages are central to antimicrobial defense, tissue repair, and immune homeostasis, broad suppression of macrophage recruitment or survival may increase infection risk and impair wound healing in immunosuppressed recipients. Moreover, many current strategies lack specificity for pathogenic intragraft macrophage subsets and may inadvertently affect beneficial systemic monocytes and tissue-resident macrophages, limiting therapeutic precision. Advances in subset-selective targeting and localized delivery may mitigate these risks but remain largely experimental and require further validation [38]. Researchers are exploring this from multiple angles:

Advanced immunosuppressive agents such as JAK inhibitors (e.g., Tofacitinib) aim to block cytokine signaling pathways that activate immune cells, including macrophages, without the broad side effects of older drugs. Costimulatory blockers such as Belatacept interrupt the T cell activation process that macrophages help sustain. Even monoclonal antibodies like Eculizumab are being used to target the complement system, another pathway that fuels rejection [39].

Immune tolerance strategies are also on the table. Regulatory T cells (Tregs) can be expanded ex vivo and infused into patients to promote graft acceptance. With CRISPR gene editing, scientists are even exploring ways to make “universal” Tregs that work regardless of donor–recipient match.

Macrophage-focused therapies directly tackle the problem cells. Drugs like CSF-1R inhibitors (Emactuzumab) reduce macrophage recruitment, while P2X7 receptor antagonists block inflammatory signaling. ROCK pathway inhibitors, such as Fasudil, are being studied to limit macrophage-driven fibrosis [40].

Beyond drugs, exosome-based therapies harness naturally occurring cellular packages to deliver immunosuppressive molecules directly to their targets, potentially avoiding systemic side effects.

Gene therapy could reprogram the immune system itself, for example, by introducing IL-10 genes to dampen inflammation [41].

**Figure 5:**
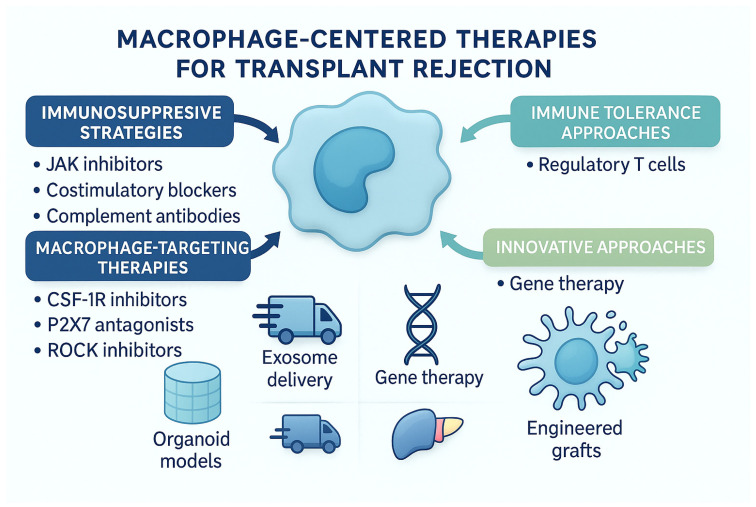
This figure summarizes emerging therapeutic strategies targeting macrophages. Traditional immunosuppressive approaches, such as JAK inhibitors, costimulatory blockers, and complement antibodies, aim to dampen upstream immune activation. More targeted therapies, including CSF-1R inhibitors, P2X7 antagonists, and ROCK inhibitors, directly modulate macrophage recruitment and inflammatory signaling. The diagram also highlights innovative tools such as exosome delivery, gene therapy, organoid-based modeling, and engineered grafts, as well as immune tolerance approaches centered on regulatory T cells. Together, these strategies illustrate the expanding therapeutic landscape aimed at reshaping macrophage behavior to improve long-term transplant outcomes.

In the lab, organoid models of human organs and AI-driven predictive tools are helping researchers anticipate rejection before symptoms appear. Furthermore, in the future, engineered grafts coated with immune-modulating molecules might arrive ready to resist rejection from day one [42] (Figure 5).

Importantly, these therapeutic strategies differ markedly in their stage of clinical translation. Costimulatory blockade with belatacept represents an established therapy in kidney transplantation and has demonstrated improved long-term graft function and patient survival compared with calcineurin inhibitor–based regimens, supporting its routine clinical use. In contrast, most macrophage-targeting approaches—including CSF1R inhibition to limit macrophage recruitment and differentiation, blockade of P2X7 receptor–mediated inflammatory signaling, ROCK pathway inhibition, and exosome- or gene-based delivery strategies—remain largely preclinical or in early-phase clinical development, with evidence derived predominantly from experimental models [43]. While these emerging strategies show promise by selectively modulating macrophage polarization and effector functions, their long-term safety, efficacy, and translational feasibility in human transplantation remain to be established (Table 1).

## 9. Conclusions

Macrophages are not just bystanders in chronic rejection; they are conductors in the immune orchestra, capable of both harmony and discord. Understanding how they switch between protective and destructive roles is opening doors to therapies that could one day keep transplanted organs working for decades. For patients, that would mean not just surviving transplantation but truly living afterward. Macrophage-focused therapeutic strategies that show efficacy in rodent transplant models face substantial translational hurdles when advancing to human trials. Fundamental differences in macrophage ontogeny, polarization programs, and immune regulation between mice and humans limit direct extrapolation of preclinical findings, particularly in the context of chronic rejection, which is difficult to faithfully model in short-lived experimental systems [44]. In addition, rodent studies often rely on simplified injury models and uniform genetic backgrounds that do not capture the heterogeneity, comorbidities, and prolonged immunosuppression characteristic of human transplant recipients. These limitations underscore the need for complementary human-based approaches, including single-cell profiling of transplant biopsies and early-phase translational studies, to bridge the gap between experimental promise and clinical applicability [45]. Finally, artificial intelligence and machine-learning frameworks are increasingly applied to integrate clinical data, histopathology, and high-dimensional omics profiles, enabling improved prediction of rejection risk and supporting personalized immunosuppressive strategies. Together, these innovations are expected to accelerate the transition from descriptive immunology toward precision-guided interventions that improve long-term graft survival.

## Figures and Tables

**Table 1:** Overview of current and emerging therapeutic approaches modulating macrophage activity in transplantation.

Therapeutic Category	Target/Mechanism	Representative Approaches	Intended Immunological Effect
**Immunosuppressive strategies**	Inhibition of intracellular inflammatory signaling	JAK inhibitors	Suppression of macrophage activation and cytokine production
	Blockade of costimulatory pathways	Costimulatory blockers	Reduced T-cell–macrophage crosstalk
	Complement pathway inhibition	Complement antibodies	Attenuation of antibody-mediated injury and inflammation
**Macrophage-targeting therapies**	Inhibition of macrophage survival and differentiation	CSF-1R inhibitors	Reduction in graft-infiltrating macrophages
	Blockade of purinergic signaling	P2X7 antagonists	Inhibition of inflammasome activation and IL-1β release
	Modulation of cytoskeletal and signaling pathways	ROCK inhibitors	Suppression of macrophage migration and pro-inflammatory polarization
**Immune tolerance approaches**	Expansion of regulatory immune networks	Regulatory T cells (Tregs)	Promotion of long-term graft tolerance
**Innovative approaches**	Targeted intercellular communication	Exosome-based delivery	Selective modulation of macrophage phenotype
	Genetic reprogramming	Gene therapy	Stable induction of anti-inflammatory macrophage states
	Advanced tissue modeling	Organoid models	Mechanistic evaluation of macrophage–graft interactions
	Bioengineered tissues	Engineered grafts	Improved graft integration and immune compatibility

## Data Availability

No new data were created or analyzed in this study. Data sharing is not applicable to this article.

## Abbreviations

α-SMA	Alpha–smooth muscle actin
AI	Artificial intelligence
AP-1	Activator protein-1
Arg1	Arginase-1
ATP	Adenosine triphosphate
CARD9	Caspase recruitment domain–containing protein 9
C/EBP	CCAAT/enhancer-binding protein
CCL2	C-C motif chemokine ligand 2
CD40	Cluster of differentiation 40
CD80	Cluster of differentiation 80
CD86	Cluster of differentiation 86
CD163	Cluster of differentiation 163
CD206	Cluster of differentiation 206
CD209	Cluster of differentiation 209 (DC-SIGN)
Clec2d	C-type lectin domain family 2 member D
CNRS	Centre National de la Recherche Scientifique
CSF1R	Colony-stimulating factor 1 receptor
CXCL10	C-X-C motif chemokine ligand 10
DAMPs	Damage-associated molecular patterns
DNGR-1	Dendritic cell NK lectin group receptor-1 (CLEC9A)
ECM	Extracellular matrix
HIF-1α	Hypoxia-inducible factor 1-alpha
HMGB1	High mobility group box 1
IKK	IκB kinase
IL-1β	Interleukin-1 beta
IL-4	Interleukin-4
IL-6	Interleukin-6
IL-10	Interleukin-10
IL-12	Interleukin-12
IL-13	Interleukin-13
IL-18	Interleukin-18
IL-23	Interleukin-23
IFN-γ	Interferon gamma
JAK	Janus kinase
KRAS	Kirsten rat sarcoma viral oncogene
MAPK	Mitogen-activated protein kinase
MCP-1	Monocyte chemoattractant protein-1 (same as CCL2)
MHC II	Major histocompatibility complex class II
miR-155	MicroRNA-155
miR-146b	MicroRNA-146b
miR-223	MicroRNA-223
MMPs	Matrix metalloproteinases
MSC	Mesenchymal stem cell
M0/M1/M2	Macrophage phenotypes (naïve, inflammatory, reparative)
NF-κB	Nuclear factor kappa-light-chain-enhancer of activated B cells
NLRP3	NOD-like receptor protein 3
NOS2	Nitric oxide synthase 2 (iNOS)
OXPHOS	Oxidative phosphorylation
P2X7	Purinergic receptor P2X ligand-gated ion channel 7
PDGF	Platelet-derived growth factor
PI3K	Phosphoinositide 3-kinase
RAGE	Receptor for advanced glycation end products
ROS	Reactive oxygen species
ROCK	Rho-associated protein kinase
SOCS	Suppressor of cytokine signaling
STAT1/STAT3/STAT6	Signal transducer and activator of transcription 1, 3, 6
TGF-β	Transforming growth factor beta
TLR2/TLR3/TLR4	Toll-like receptors 2, 3, 4
TNF-α	Tumor necrosis factor alpha
TRIF	TIR-domain-containing adapter-inducing interferon-β
VEGF	Vascular endothelial growth factor
